# Identification of necroptosis-related gene TRAF5 as potential target of diagnosing atherosclerosis and assessing its stability

**DOI:** 10.1186/s12920-023-01573-0

**Published:** 2023-06-17

**Authors:** Zhanli Peng, Kangjie Wang, Shenming Wang, Ridong Wu, Chen Yao

**Affiliations:** 1 Division of Vascular Surgery, The First Affiliated Hospital, Sun Yat-Sen University, Guangzhou, China; 2 National-Guangdong Joint Engineering Laboratory for Diagnosis and Treatment of Vascular Diseases, First Affiliated Hospital, Sun Yat-Sen University, Guangzhou, China

**Keywords:** Atherosclerosis, Necroptosis, *TRAF5*, Diagnose, Stability

## Abstract

**Background:**

Atherosclerosis (AS) is a leading cause of morbidity and mortality in older patients and features progressive formation of plaques in vascular tissues. With the progression of atherosclerosis, plaque rupture may occur and cause stroke, myocardial infarction, etc. Different forms of cell death promote the formation of a necrotic core of the plaque, leading to rupture. Necroptosis is a type of programmed cell death that contributes to the development of cardiovascular disease. However, the role of necroptosis in AS has not yet been investigated.

**Methods:**

The Gene Expression Omnibus (GEO) database was used to obtain gene expression profiles. Differentially expressed genes (DEGs) and necroptosis gene sets were used to identify necroptosis-related differentially expressed genes (NRDEGs). The NRDEGs were used to construct a diagnostic model and were further screened using least absolute shrinkage selection operator (LASSO) regression and random forest (RF) analysis. The discriminatory capacity of the NRDEGs was evaluated using receiver operating characteristic (ROC) curves. Immune infiltration levels were estimated based on CIBERSORTx analysis. The GSE21545 dataset, containing survival information, was used to determine prognosis-associated genes. Univariate and multivariate Cox regression analyses combined with survival analysis determined gene prognostic values. RNA and protein levels were detected by RT-qPCR and western blotting in arteriosclerosis obliterans(ASO) and normal vascular tissues. Vascular smooth muscle cells (VSMCs) were treated with oxidized low-density lipoprotein (ox-LDL) to develop cell models of advanced AS. The effects of protein knockdown on necroptosis were assessed by western blotting and flow cytometry. EdU and Cell Counting Kit-8 assays were used to examine cell proliferation.

**Results:**

TNF Receptor Associated Factor 5 (*TRAF5)* was identified as a diagnostic marker for AS based on the AUC value in both the GSE20129 and GSE43292 datasets. According to differential expression analysis, LASSO regression analysis, RF analysis, univariate analysis, multivariate analysis, and gene-level survival analysis, *TRAF5* was markedly associated with necroptosis in AS. Silencing *TRAF5* promotes necroptosis and attenuates the proliferation of ox-LDL-induced cell models of advanced AS.

**Conclusions:**

This study identified a diagnostic marker of necroptosis-related atherosclerosis, *TRAF5*, which can also be used to diagnose and assess atherosclerotic plaque stability. This novel finding has important implications in the diagnosis and assessment of plaque stability in atherosclerosis.

**Supplementary Information:**

The online version contains supplementary material available at 10.1186/s12920-023-01573-0.

## Introduction

Atherosclerosis (AS) is a major cause of mortality and morbidity among middle-aged and older patients [1]. It is often the most common underlying cause of cerebrovascular, peripheral vascular, and coronary heart diseases. AS is often considered a stable, chronic disease. However, plaque rupture often results in severe acute ischemic events [2]. With AS progression, a necrotic core develops at the center of the plaque. The different mechanisms of programmed death in foam cells and vascular smooth muscle cells are thought to be important causes of necrotic core development [3, 4]. Early lesions show low levels of cell death but appear with increasing frequency in the necrotic core and fibrous cap [5]. In advanced plaques, cell death outpaces cell proliferation [6], which suggests that necroptosis is critical to our understanding of necrotic core formation.

Necroptosis, a form of programmed cell death, is characterized by both necrosis and apoptosis [7] and is considered an important pathway in a variety of diseases. The necroptosis pathway is often triggered by various signals, including the death receptor ligands INF-α and the TNF superfamily, causing organelle swelling, loss of cell membrane integrity, cell disruption, and extravasation of cell contents [6, 8]. The activated signaling pathway causes MLKL phosphorylation to form oligomers and translocate to the cell membrane causing cell rupture and release of cell contents triggering a series of inflammatory responses [9]. Recently, a variety of studies have explored necroptosis mechanisms in the cardiovascular domain [10–13]. For instance, Karunakaran et al. found that the necroptosis inhibitor Nec-1 reduces the necrotic core area and markers of plaque instability [11]. Leeper similarly emphasized the role of necroptosis in atherosclerosis [14]. However, this phenomenon has not yet been empirically confirmed. To date, few studies have explored the relationship between necroptosis-related genes and AS.

Herein, we downloaded datasets from the GEO and conducted additional bioinformatics analysis to determine necroptotic-associated genes in AS. Afterward, we confirmed our results in patient-derived vascular smooth muscle cells (VSMCs) treated with oxidized low-density lipoprotein (ox-LDL). Our results identified a protein that has prognostic strength and can promote necroptosis in VSMCs, which will inform future studies in AS.

## Methods

### Data capturing

**Fig. 1:**
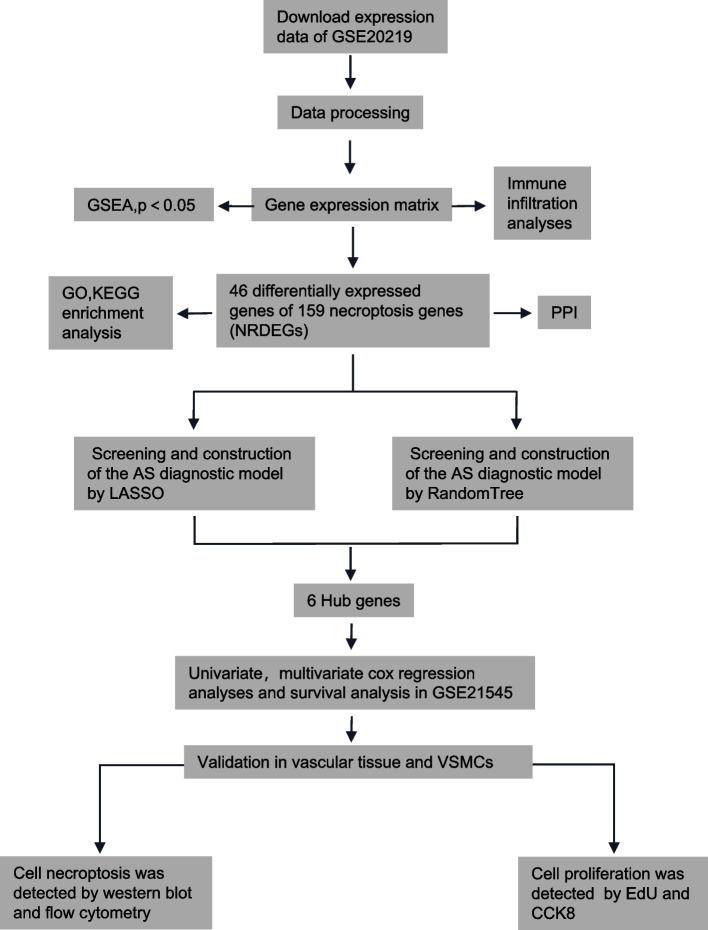
The workflow implemented in this study

A collection of 86 non-AS and 49 AS mRNA expression profiles from venous blood was obtained from the GSE20129 dataset and used as the training set [15]. Thirty-two pairs of atheromatous plaque and control samples were curated from the GSE43292 dataset as a validation set [16]. The GSE21545 dataset includes 97 peripheral blood samples taken from patients that underwent endarterectomy and their transcriptomic profiling, follow-up, and survival information [17]. The microarray dataset GSE23303 included data from three smooth muscle cell-enriched sections, three macrophage-enriched sections, and three whole sections from carotid plaque [18].Twelve control and 29 AS carotid arteries were retrieved from the GSE100927 dataset [19]. All datasets were obtained from the GEO repository (https://www.ncbi.nlm.nih.gov/gds/). The corresponding probe annotation files were used for gene-symbol conversion. A total of 159 necroptosis-related genes were downloaded from the Kyoto Encyclopedia of Genes and Genomes (KEGG) pathway database. The Fig. 1 depicts a process.

### Identification of differentially expressed necroptosis-related genes

Reannotation of the series matrix was performed using the “AnnoProbe” package. To calibrate the microarray data and identify the differentially expressed genes (DEGs) between AS and control samples, the “limma” package in the R software was used [20]. mRNAs that met the defined criteria (*p* value < 0.05) were considered DEGs. Thereafter, necroptosis-related differentially expressed genes (NRDEGs) were defined as the intersection of DEGs and necroptosis-related genes (NRGs). The “VennDiagram” package was used to visualize the number of NRDEGs. The “ggplot2” package was used to visualize NRDEGs expressions in a heatmap.

### Protein–protein interaction analysis

The interactions of the various NRDEGs were examined using the STRING database (http://string-db.org/). Then, the protein–protein interaction (PPI) network was built and visualized using Cytoscape software version 3.9.1 (http://cytoscape.org/) [21].

### Function enrichment analysis

The “ClusterProfiler” package was used to perform Gene Ontology (GO) and KEGG pathway enrichment analyses(www.kegg.jp/kegg/kegg1.html) for AS-specific NRGs [22–25]. There was a significant enrichment of terms with FDR of < 0.05. Gene set enrichment analysis (GSEA) was performed using the “clusterProfiler” package to find the atherosclerotic-related signaling pathways of c2 (c2.cp.kegg.v7.5.1. entrez.gmt) and c5 (c5.bp.v7.5.1. entrez. gmt) in the molecular signature database [22]. A nominal *p* value < 0.05 was considered as a statistically significant gene set.

### Screening and construction of necroptosis-related gene signatures for AS diagnosis

The least absolute shrinkage selection operator (LASSO) regression analysis algorithm was used to reduce the risk of overfitting and enhance forecast precision. The “glmnet” software package was used to identify genes that could be used to discriminate between AS and non-AS specimens and significantly different results. Ten-fold cross-validation was used to optimize parameter selection. Lambda was considered as the minimum partial likelihood of deviance. Receiver operating characteristic (ROC) curves were generated to verify the predictive significance of the identified NRG signature [26]. To determine diagnostic accuracy, the area under the ROC curve (AUC) was calculated. The random forest (RF) model was used to evaluate the importance of NRDEGs with 800 trees. Of all samples, 70% of the samples were randomly selected for the training set, and the others were selected as the test set. RF analysis was constructed using the “RandomForest” package [27]. ROC curves were used to verify the predictive significance of this model [26]. Kaplan–Meier survival curves were drawn using the “survival” package. Cox proportional hazards regression analysis was used to perform the univariate and multivariate survival analyses.

### Immune infiltration analysis and correlation between identified mRNA and immune cells

Based on the LM22 immunological signature gene set, the immune landscape was evaluated using the CIBERSORTx website analysis tool (https://cibersortx.stanford.edu/) [28]. Pearson’s correlation coefficient analysis was used to calculate the association between the identified mRNAs and immune cells.

### Cell extraction, isolation, culture, and treatment

Three arteriosclerosis obliterans (ASO) samples were obtained from individuals with ASO suffering from severe lower-limb ischemia. Following amputation, the superficial femoral arteries were divided. Three healthy donors with no history of ASO or arteriostenosis were selected for normal artery acquisition. Following superficial femoral artery separation, only arteries with normal vascular tissue were preserved for subsequent studies.

Normal renal arterial tissues were obtained from healthy organ donors and used for VSMCs culture. VSMCs were prepared using the established explant method and identified by staining with an anti-smooth muscle α-actin antibody. Briefly, VSMCs were maintained in DMEM (Gibco, Carlsbad, CA, USA) supplemented with 10% Australian Special fetal bovine serum at 37 °C in a humidified 5% CO2 incubator. Cells from the third to fifth passage were used in this study. VSMCs were induced with 100 mg/L ox-LDL (Yiyuan Biotechnologies, Guangzhou, China) for 48 h to develop cell models of advanced AS lesions.

### Cell transfection

Anti-*TRAF5* small interfering RNA (si-*TRAF5*) and siRNA negative control (si-NC) were synthesized by Tsingke Biotechnology Co., Ltd (Guangzhou, China). VSMCs were transfected using Lipofectamine RNAiMAX reagent (Invitrogen, Carlsbad, CA, USA) for 48 h. Reverse transcription-quantitative polymerase chain reaction (RT-qPCR) was used to assess the effectiveness of the si-*TRAF5* transfection. The sequences of the siRNA used in this study are shown in Supplementary Table 1.

### Quantitative reverse-transcription quantitative-polymerase chain reaction and tissue acquisition

Total RNA was extracted from VSMCs and arterial specimens using the AG RNAex Pro Reagent (Hunan Accurate Biology Co., Ltd, AG21102, Hunan, China). A Nanodrop 2000 was used to measure the RNA concentration. The Evo M-MLV Mix Kit (Hunan Accurate Biology, AG11728) was used to reverse transcribe RNA into cDNA, according to the manufacturer’s guidelines. RT-qPCR was performed on a LightCycler 480 (Roche, Basel, Switzerland) using the SYBR Green Premix Pro Taq HS qPCR Kit (Hunan Accurate Biology, AG11701). The expression of target genes was estimated using the 2 − ΔΔCt approach, with GAPDH as a reference control. Primer information is provided in the Supplementary Table 2.

### Western blotting

Six-well plates containing VSMCs were seeded and cultured to 70% confluency. Cells were lysed on ice using RIPA buffer, and 20 μg protein was separated by SDS-PAGE then transferred to a PVDF membrane. The membrane was blocked for 1 h at room temperature using Tris-buffered saline and Tween-20 (TBST) and 5% nonfat milk powder. The membrane was then incubated with primary antibodies against RIPK3 (1:3000; 17,563–1-AP; Proteintech Group, Inc., Wuhan, China), MLKL (1:800; 21,066–1-AP; Proteintech Group), and phospho-MLKL (S358) (1:800; T57146; Abmart Pharmaceutical Co., Ltd, Shanghai, China) at 4 °C overnight. The membrane was then washed three times with TBST and incubated for 1 h at room temperature with HRP-conjugated goat anti-rabbit or anti-mouse antibody (1:9000; SA00001-2 or SA00001-1, respectively; Proteintech Group). Immunoreactivity was measured using an improved chemiluminescence detection method. An automatic digital gel image analysis system was used to capture the images.

### Indirect immunofluorescence double staining method

Indirect immunofluorescence double staining method was performed to visualize *TRAF5* and *SM22* expression in ASO tissue frozen section using the primary antibodies rabbit *TRAF5* monoclonal antibody (1:50, ab303522, Abcam, Cambridge, UK), mouse SM22 monoclonal antibody (1:3000, 60,213–1-Ig; Proteintech Group, Inc., Wuhan, China). We permeabilized the sections with Triton X-100 (0.1%) for 30 min at 4 °C, washed with PBS(1X) and blocked with 5% Fetal bovine serum in PBS for 1 h. The primary antibodies were added to the blocking solution and incubated at 4 °C overnight on an orbital shaker. Next day, following three washings with PBS, the sections were incubated in blocking solution containing the secondary antibodies [CoraLite594 – conjugated Goat Anti-Rabbit IgG(H + L) (1:100; SA00013-4; Proteintech Group, Inc., Wuhan, China) and CoraLite488-conjugated Goat Anti-Mouse IgG(H + L) (1:100, SA00013-1; Proteintech Group, Inc., Wuhan, China)] for 1 h at room temperature. After final washes with PBS three times, we used DAPI to stain the nucleus for 10 min and finally put the anti-fluorescence attenuation sealant( MIKX, DB255, Shenzhen, China) onto a glass slide and cover with a cover glass slip. The sections were examined using a DMi8 microscope (Leica Microsystems).

### Flow cytometric analysis

Cell death was evaluated using an Annexin V-APC/PI staining kit (Nanjing KeyGen Biotech Co., Ltd, Nanjing, China) according to the manufacturer’s instructions. Briefly, VSMCs were collected by digestion with EDTA-free trypsin, made into single-cell suspensions, incubated with Annexin V-APC, and analyzed using a Beckman Coulter CytoFLEX flow cytometer.

#### CCK8 assay

Cell proliferation was detected using the Cell Counting Kit-8 (CCK8) colorimetric assay (CK04-20; Dojindo Laboratories, Kumamoto, Japan), according to the manufacturer’s instructions. VSMCs were then cultured in 96-well plates and 10 μL CCK8 was added to each well at the indicated times. These plates were further incubated for 4 h, and the medium was replaced with 150 μL of DMSO. The absorbance was measured at 450 nm.

#### EdU assay

EdU staining was performed following the manufacturer’s instructions using the BeyoClick EdU Cell Proliferation Kit (Beyotime Biotechnology, Shanghai, China). Cells were incubated with EdU for 6 h. Images were obtained using a ZEISS Axio Observer D1 inverted dynamic fluorescence microscope (Carl Zeiss AG, Oberkochen, Germany) at 100 × with the ZEN software. The percentage of EdU-positive cells (EdU positive/DAPI positive × 100) was analyzed using the ImageJ software.

### Statistical analysis

R software 4.1.2 and RStudio 2021.09.1 + 372 (Boston, USA) were used for data analysis and visualization. The comparison of gene expression between two groups was assessed by the two-tailed unpaired t-test using GraphPad Prism 9.0 (California, USA). The results were presented as means and standard deviations. Unless otherwise stated, the *p* value < 0.05 was considered statistically.

## Results

### Identification of DEGs and NRDEGs

**Fig. 2:**
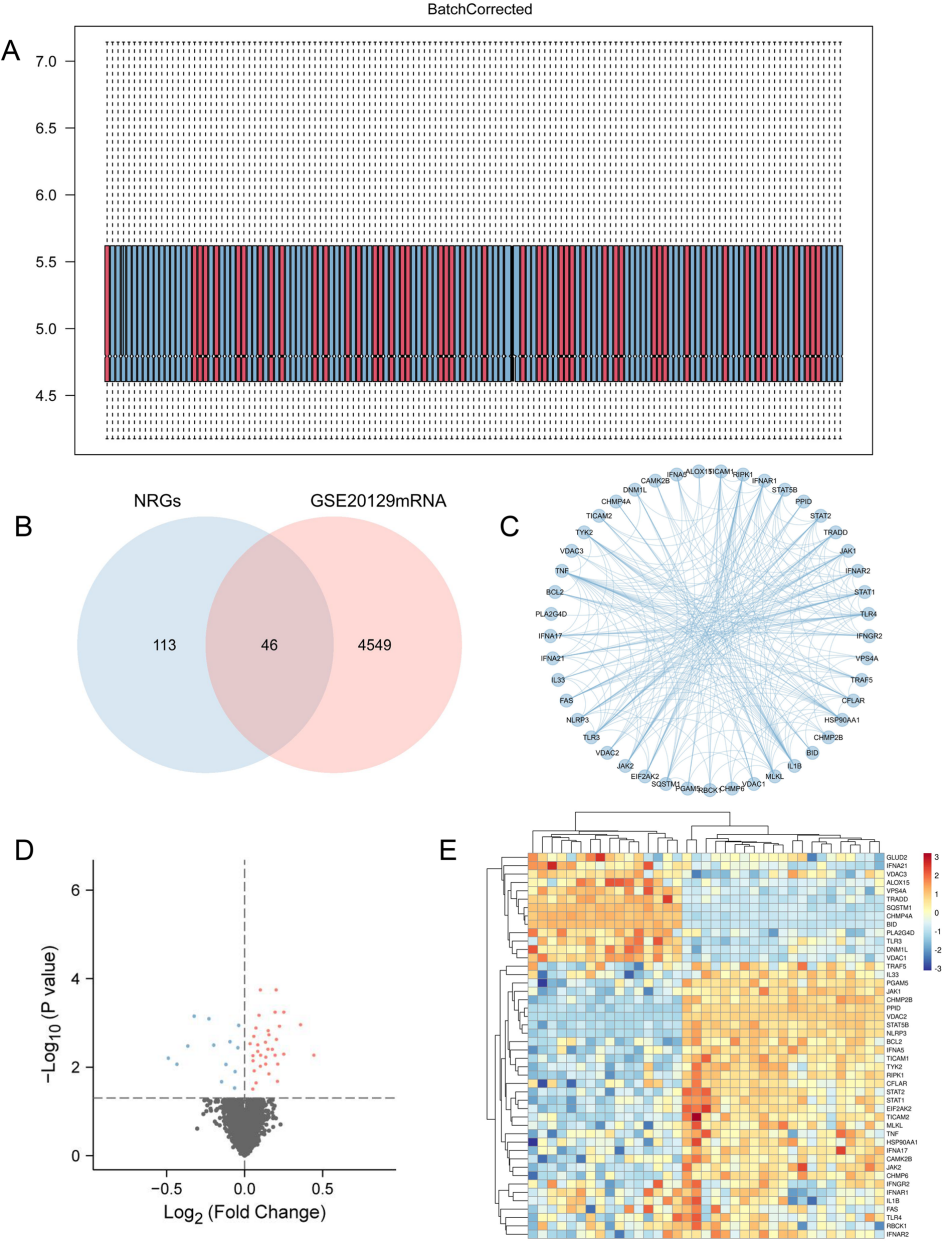
Expression patterns of necroptosis-related genes in atherosclerosis (AS). **A** Box plots showing the samples of GSE20129 after batch correction. **B** Venn diagrams display the necroptosis-related differentially expressed genes (NRDEGs). **C** The protein–protein interaction network constructed using the STRING database for NRDEGs. **D** Visualization of the expression of genes associated with necroptosis in AS and non-AS samples using volcano plots. Orange bubbles represent upregulated genes, blue bubbles represent downregulated genes, and gray bubbles represent non-significant genes, *p* < 0.05. **E** Heatmap displaying the expressions of the 46 NRDEGs. Red bricks indicate the more highly expressed NRGs and blue bricks indicate lower expression

The GSE20129 dataset was used to study gene expression in AS compared to corresponding healthy blood samples using the limma package. The batch effect was corrected using the ComBat package (Fig. 2A). In GSE20129, our study found 4595 DEGs overall, with 2387 upregulated genes and 2208 downregulated genes, compared to AS and normal blood samples. A total of 46 differentially expressed NRGs were classified as NRDEGs after taking the intersection of DEGs and NRGs (Fig. 2B). 46 AS-specific NRDEG close interactions were identified via the PPI network (Fig. 2C). Additionally, the expression volcano plot and heatmap show the NRDEGs (Fig. 2D, E).

### GSEA and functional analyses profiles

**Fig. 3:**
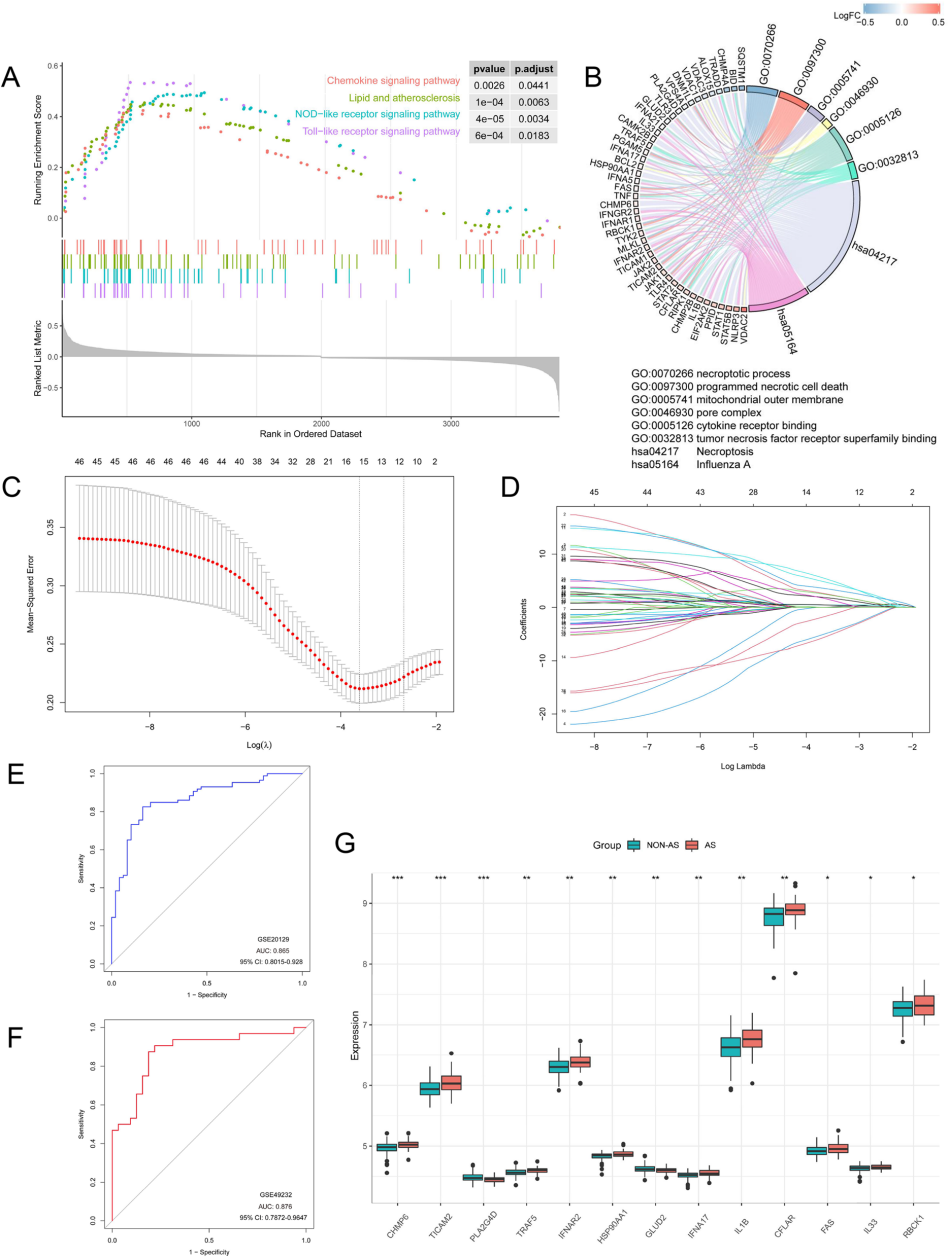
Necroptosis-related genes and their biological significance in atherosclerosis (AS) and construction of the AS diagnostic model and screening of necroptosis-related differentially expressed genes (NRDEGs) by least absolute shrinkage selection operator (LASSO) regression analysis. **A** Gene set enrichment analysis results for the activation of pathways in AS than in non-AS specimens. **B** Biological processes (BPs), molecular functions (MFs), and Kyoto Encyclopedia of Genes and Genomes pathways (www.kegg.jp/kegg/kegg1.html) associated with NRDEGs. **C** Tuning feature selection in the LASSO model. **D** Profiles of the LASSO regression coefficients. **E** Receiver operating characteristic (ROC) curves for assessing the diagnostic effectiveness of the LASSO model in GSE20129 (training set). **F** ROC curves for assessing the LASSO model's diagnostic performance in GSE43292 (testing set). **G** Box plots showing the 13 NRDEGs after screening by the LASSO method. (*p < 0.05; **p < 0.01; ***p < 0.001)

We identified the predominant signaling pathways between AS and non-AS specimens using the GSEA method. Our results revealed that the lipid and AS, NOD-like receptor, Toll-like receptor, and chemokine signaling pathways exhibited increased activation in AS samples (Fig. 3A), FDR < 0.05 (Supplementary Table 3). The functions of the NRDEGs and linked pathways were determined using GO and KEGG enrichment analyses. GO revealed enrichment of the necroptotic process, programmed necrotic cell death, mitochondrial outer membrane regulation, pore complex regulation, cytokine receptor binding, and tumor necrosis factor receptor superfamily binding. KEGG enrichment analysis of NRDEGs revealed the enrichment of necroptosis and influenza A (Fig. 3B), FDR < 0.05 (Supplementary Table 4). These results suggest that necroptotic and immune biological pathways and mechanisms may contribute to AS development.

### Screening and creation of AS diagnostic NRG signatures

**Fig. 4:**
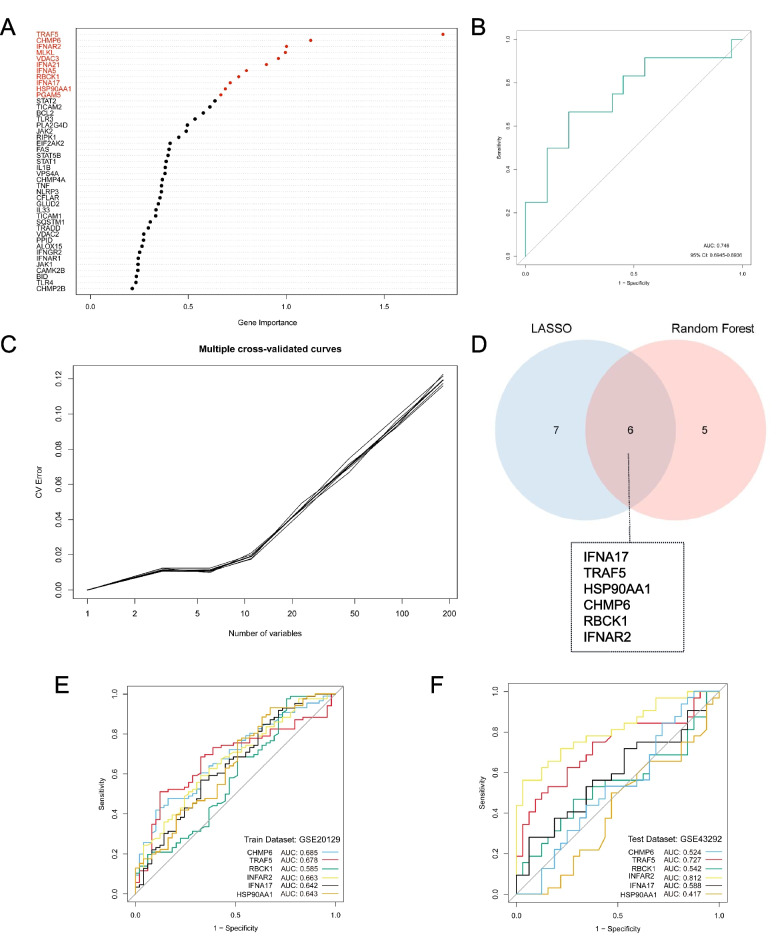
Screening the necroptosis-related differentially expressed genes (NRDEGs) by the random forest (RF) method and intersecting with least absolute shrinkage selection operator (LASSO)-screened genes to identify hub-NRDEGs. **A** Gene importance ranking and selection of the top 11 genes based on cross-validation error rate curves. **B** Receiver operating correlation (ROC) curves for assessing the effectiveness of the RF model. **C** Multiple cross-validation curves were consistent. **D** 6 hub-NRDEGs overlapping in LASSO and RF methods. **E** ROC curves for rating the performance of 6 hub-NRDEGs in GSE20129 (training set). **F** ROC curves for rating the performance of 6 hub-NRDEGs in GSE43292 (testing set)

AS-specific NRDEGs were screened using LASSO analysis, and a diagnostic model containing 13 genes (*TICAM2, IFNA17, PLA2G4D, TRAF5, HSP90AA1, CHMP6, RBCK1, IFNAR2, IL1B, GLUD2, CFLAR, FAS,* and *IL33*) for AS was developed (Fig. 3C, D). Among them, *TICAM2, IFNA17, TRAF5, HSP90AA1, CHMP6, RBCK1, IFNAR2, IL1B, CFLAR, FAS,* and *IL33*, showed higher expression in AS specimens. Additionally, AS specimens had significantly lower levels of *PLA2G4D* and *GLUD2* than non-AS specimens (Fig. 3G). The AUC scores in the GSE20129 and GSE43292 datasets were 0.8665 and 0.876 (Fig. 3E, F). To ensure the stability of the model, RF analysis was used to screen the NRDEGs according to gene importance (Fig. 4A). Multiple cross-validation curves were coincident, and the AUC value was 0.746 (Fig. 4B, C). Using the cross-validation curve, the top 11 NRDEGs (*TRAF5, CHMP6, IFNAR2, MLKL, VDAC3, IFNA21, IFNA5, RBCK1, IFNA17, HSP90AA1, PGAM5*) were selected. After intersecting the LASSO and RF results, six hub-NRDEGs (*IFNA17, TRAF5, HSP90AA1, CHMP6, RBCK1,* and *IFNAR2*) were identified and selected (Fig. 4D). *TRAF5* and *INFAR2* had excellent diagnostic efficacy in both GSE20129 (*TRAF5*: AUC = 0.678; *IFNAR2*: AUC = 0.663) and GSE43292 (*TRAF5*: AUC = 0.727; *IFNAR2*: AUC = 0.812) (Fig. 4E, F).

### Analysis of hub-NRDEGs with patient survival information

**Fig. 5:**
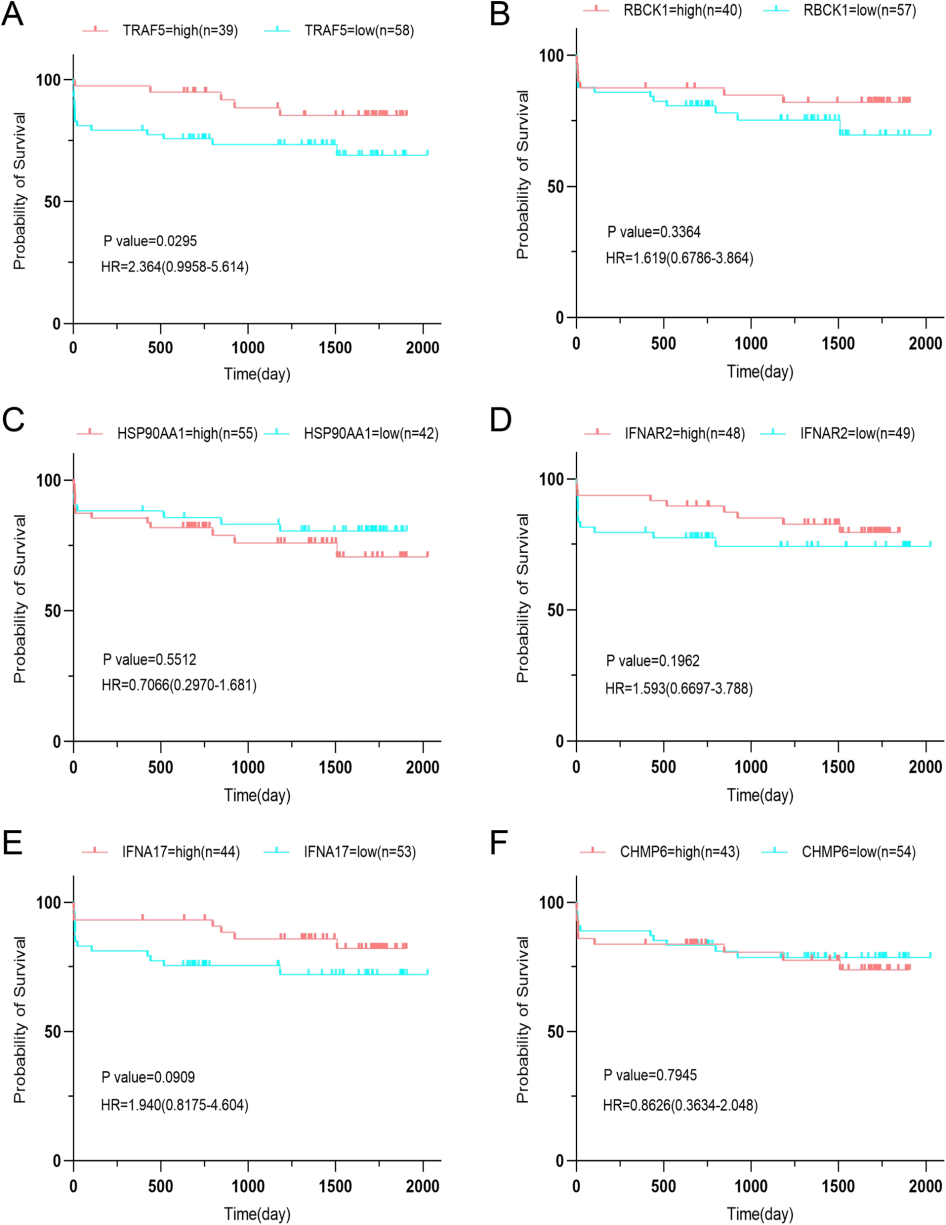
Survival analysis of patients with atherosclerosis in the GSE21545 dataset with high and low expressions of 6 hub-NRDEGs. **A** *TRAF5* **B** *RBCK1* **C** *HSP90AA1* **D** *IFNAR2* **E** *IFNA17* **F** *CHMP6*

To further evaluate the relationship between hub-NRDEGs and survival factors, we performed univariate and multivariate analyses of these genes in the GSE21545 dataset. Univariate analysis revealed that *TRAF5* and *RBCK1* met the conditions (*p* < 0.05), and further multivariate analysis was performed. Multivariate analysis revealed that *TRAF5* expression may be an independent prognostic factor (*p* < 0.05) (Table 1). Survival analysis revealed that *TRAF5* was prognostically significant in the GSE21545 dataset (Fig. 5A–F).

**Table 1:** Univariate and multivariate analysis

Characteristics	Total(N)	Univariate analysis		Multivariate analysis	
		Hazard ratio (95% CI)	*P* value	Hazard ratio (95% CI)	*P* value
*TRAF5*	97	0.012 (0.000–0.331)	0.009*	0.024 (0.001–0.925)	0.045*
*IFNAR2*	97	0.963 (0.182–5.098)	0.964		
*RBCK1*	97	0.180 (0.034–0.950)	0.043*	0.390 (0.061–2.484)	0.319
*HSP90AA1*	97	0.905 (0.160–5.118)	0.910		
*IFNA17*	97	0.350 (0.078–1.577)	0.172		
*CHMP6*	97	1.635 (0.247–10.815)	0.610		

^*^p < 0.05

### Signatures of the hub-NRDEGs linked to immune infiltration in AS

**Fig. 6:**
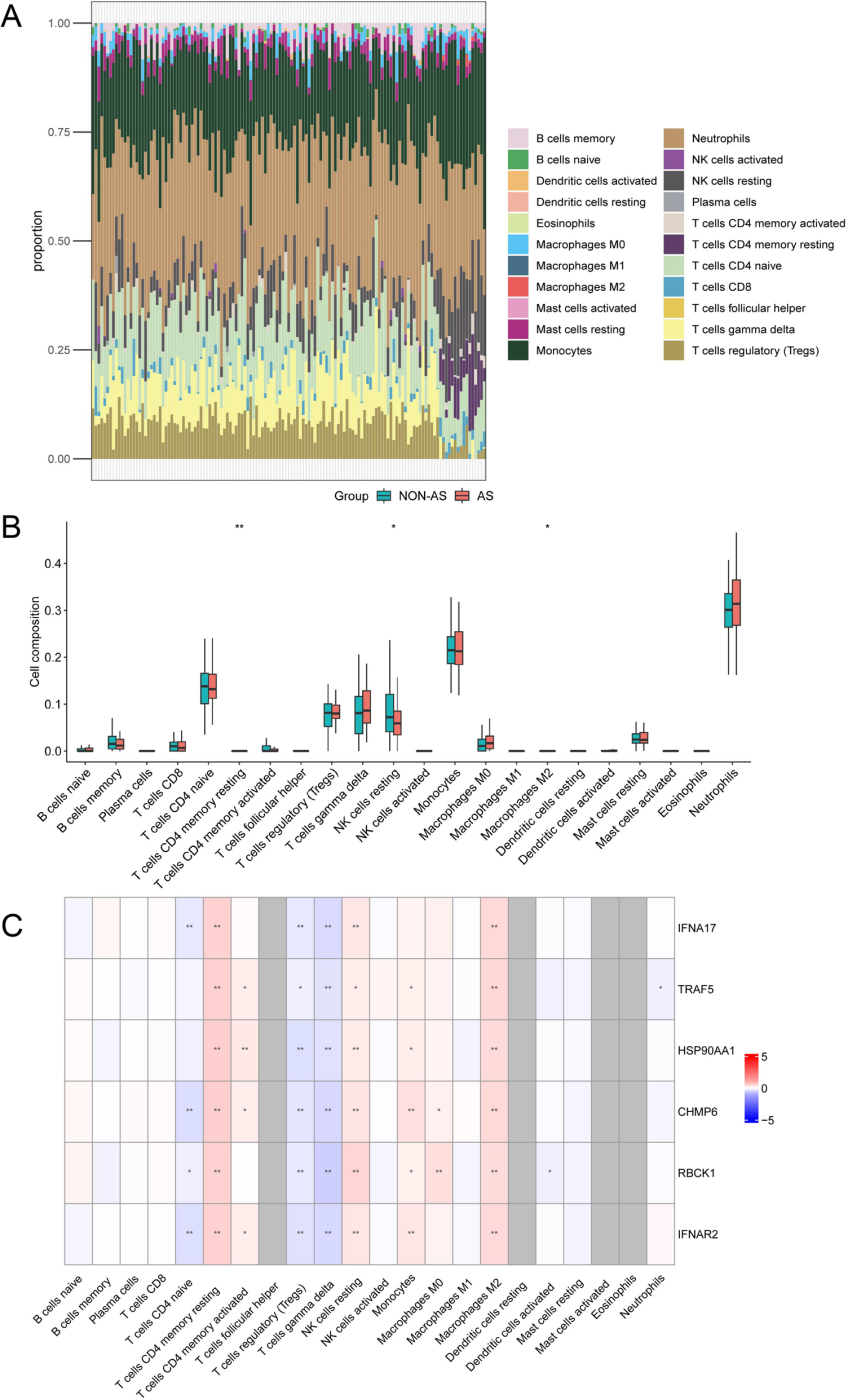
The landscape of immune infiltration in atherosclerosis. **A** Stacked bar chart of the immune cell infiltrates. **B** The box plot of the immune cell proportions. **C** Heatmaps visualizing the associations of feature 6 hub-NRDEGs with immune cell infiltrations. (*p < 0.05; **p < 0.01)

Given that both GSEA and functional enrichment results were associated with immune and inflammatory pathways, we assessed the differences in immune infiltration between AS and non-AS specimens. Figure 6A shows the landscape of immune infiltration in both groups. We discovered that non-AS blood samples had higher infiltration levels of naïve CD4 + T cells, resting NK cells, and M2 macrophages than those in the AS specimens (Fig. 6B). As shown in Fig. 6C, hub-NRDEGs were associated with immune cell infiltration. Most of these genes were positively correlated with resting memory CD4 + T cells, resting NK cells, monocytes, and M2 macrophages but were negatively associated with regulatory T cells and gamma delta T cells.

### Validation of *TRAF5* expression

**Fig. 7:**
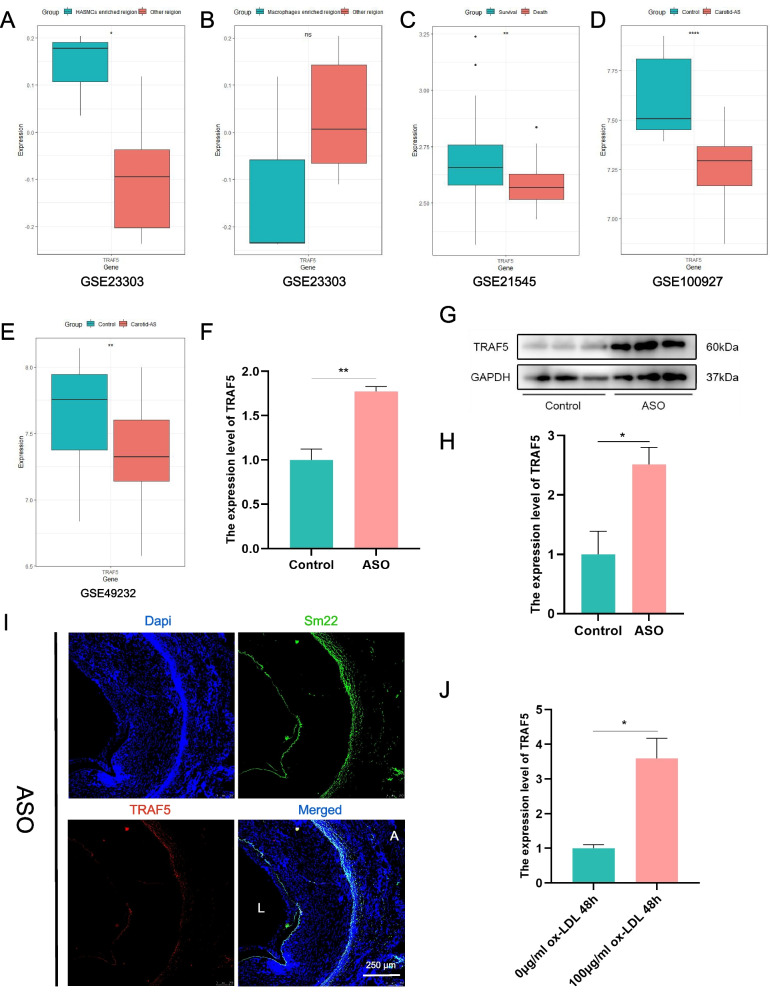
The gene expression level in the datasets, advanced atherosclerosis cell model, and arterial tissue. **A**, **B** In the GSE23303 dataset, assessment of *TRAF5* expression in smooth muscle cells and macrophage-enriched areas. **C** In the GSE21545 dataset, assessment of *TRAF5* expression in death and survival cases. **D**, **E** In the GSE100927 and GSE49232 dataset, assessment of *TRAF5* expression in carotid plaque and normal samples. **F** Validation of *TRAF5* expression in the artery samples by RT-qPCR (*n* = 3). **G**, **H** Validation of *TRAF5* expression in the artery samples by Western-blotting (*n* = 3, normalized to GAPDH). **I** The co-localization of *TRAF5* and *SM22* in ASO tissue. **J** Validation of *TRAF5* expression in VSMCs treated with 100 mg/L oxidized-low density lipoprotein (*n* = 3). (*p < 0.05; **p < 0.01; ***p < 0.001; ****p < 0.0001)

Subsequently, we validated the expression of these hub-NRDEGs in additional datasets. In the GSE23303 dataset, we found that *TRAF5* expression in smooth muscle cell-enriched regions was higher than that in other regions (Fig. 7A, B). We also found the co-localization of *TRAF5* and *SM22*(A smooth muscle cell-specific marker) in ASO tissue through the indirect immunofluorescence double staining method (Fig. 7I). The level of *TRAF5* expression was markedly increased in the surviving cases compared to the deceased cases in the GSE21545 dataset (Fig. 7C). In both the GSE100927 and GSE43292 datasets, we found that *TRAF5* exhibited distinct downregulation in AS carotid samples (Fig. 7D, E). We further validated *TRAF5* expression in femoral artery tissues and VSMCs. We discovered that ASO tissues had higher levels of *TRAF5* expression than normal arteries at the RNA and protein level (Fig. 7F, G-H). When VSMCs were treated with ox-LDL, *TRAF5* expression was significantly higher than that in untreated VSMCs (Fig. 7J), which is consistent. These results suggest that *TRAF5* is involved in the development of AS and is active in VSMCs.

### *TRAF5* knockdown enhances necroptosis and attenuates proliferation of ox-LDL-induced VSMCs

**Fig. 8:**
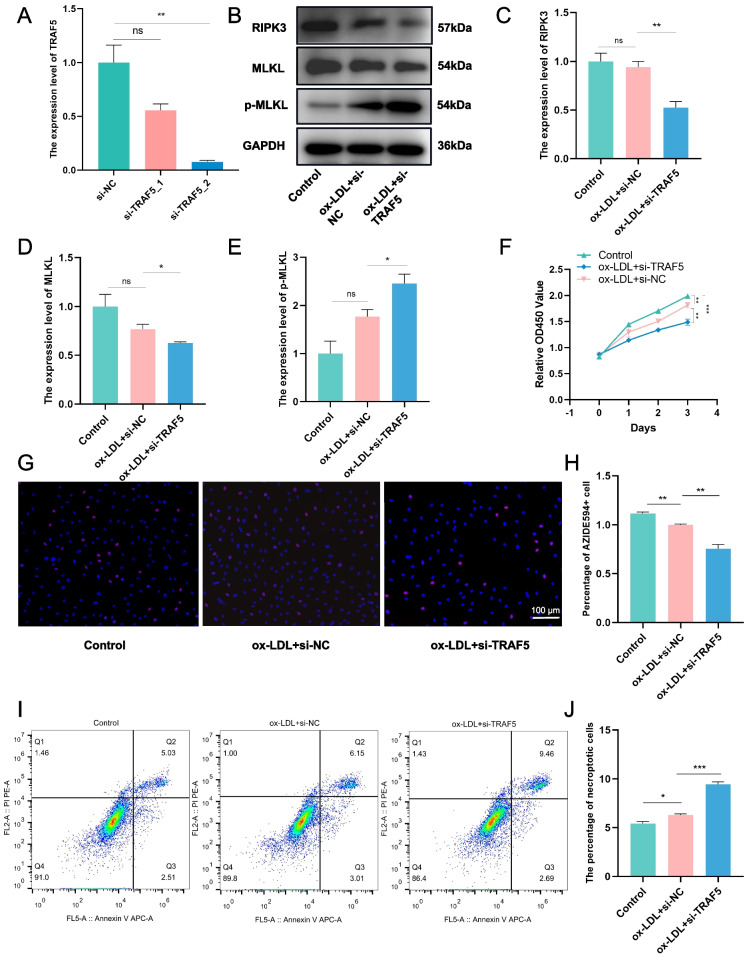
*TRAF5* knockdown enhances necroptosis and attenuates the proliferation of VSMCs induced by oxidized-low density lipoprotein (ox-LDL). **A** RT-qPCR to confirming *TRAF5* expression in VSMCs treated with *TRAF5* siRNAs. **B**-**E** Western blotting detecting the levels of RIPK3, MLKL, and p-MLKL in ox-LDL-induced VSMCs with *TRAF5* knockdown (*n* = 3, normalized to GAPDH). **F** Cell counting kit 8 assay (CCK8) evaluating cell proliferation (*n* = 3). **G**, **H** EdU assay evaluating cell proliferation (*n* = 3). **I**, **J** Necroptosis was detected by flow cytometry after Annexin V/PI staining. Annexin V-/PI- represents living cells, Annexin V + /PI- represents early necroptotic cells, and Annexin V + /PI + represents necroptotic cells (*n* = 3). (ns: no significance; *p < 0.05; **p < 0.01; ***p < 0.001)

We used siRNA to decrease *TRAF5* levels in VSMCs to examine its function (Fig. 8A). We also induced advanced AS cellular models by exposing VSMCs to ox-LDL. We detected RIPK3, MLKL, and p-MLKL, factors in the necroptosis signaling pathway, by western blotting [29–31]. *TRAF5* knockdown decreased RIPK3 and MLKL and increased p-MLKL levels (Fig. 8B–E), which means that the necroptosis pathway activated. VSMC proliferation was associated with plaque stability. After *TRAF5* knockdown, we evaluated the proliferation of VSMCs using EdU and CCK8 assays, which revealed that *TRAF5* knockdown attenuated proliferation (Fig. 8F, G–H). Flow cytometry results showed that *TRAF5* knockdown increased the proportion of necroptotic VSMCs (Fig. 8I, J).

## Discussion

Necroptosis is involved in several cardiovascular and cerebrovascular diseases [32–35]. Understanding the mechanism by which necroptosis causes plaque destabilization is of great importance to discover new treatment strategies for acute ischemic events. This study provides an assessment of necroptosis-related genes in patients with AS using differential expression analysis. According to GO, KEGG, and GSEA results, NRDEGs specific to AS were shown to be involved in regulating biological processes, including the necroptotic process, AS-related, and immunity-related signaling pathways, which indicated that AS-specific necroptosis-related genes play a significant role in AS pathogenesis. We also showed that *TRAF5* is an independent diagnostic marker of AS based on LASSO and RF analyses. The strong diagnostic capability of AS was demonstrated using ROC curves. Because AS is a chronic, systemic inflammatory illness characterized by an activated innate immune response [36], we performed immune infiltration analysis and found a strong correlation between M2-type macrophages and hub-NRDEGs. Next, we analyzed the expression of hub-NRDEGs in different cell-enriched regions of the arterial wall in the GSE23303 dataset, we found that only *TRAF5* expression was statistically different in the smooth muscle cell-enriched layer. Through the co-localization of *TRAF5* and *SM22* in ASO tissue, we found that *TRAF5* located in the smooth muscle cell layer. These results further suggested that *TRAF5* is prognostically relevant and plays a role in smooth muscle cell biology.

The role of foam cells in AS has been widely reported, but there are few studies on macrophage-like smooth muscle cell-derived foam cells. Previously, the conversion of VSMCs to macrophage-like foam cells was reported to be driven by lipid accumulation in the plaques [37]. The gene expression of these cells is markedly different from that of traditional macrophages [38] and their phagocytic capability is lower than that of activated peritoneal macrophages. In advanced AS, at least 50% of foam cells are VSMC-derived [39], and reduced phagocytosis combined with a high proportion of macrophage-like smooth muscle cells can directly contribute to the development of the plaque necrotic core [40]. Necroptosis, a mode of cell death, has rarely been reported in this field. Wang et al. reported that enhanced RIPK3 signaling causes smooth muscle cell necroptosis in aneurysmal tissues [41]. In advanced atherosclerotic plaques, necroptosis is activated [13, 42] and is characterized by the development of atheromatous plaques with fibrous caps and necrotic cores. Thus, VSMC necroptosis may lead to plaque instability [43]. *TRAF5* is an intracellular signaling bridging protein associated with lymphotoxin β and CD40 receptors that mediate protective downstream signaling in inflammation [44, 45]. *TRAF5* deficiency accelerates the recruitment of inflammatory cells and foam cell formation, thereby accelerating AS formation in mice [46]. *TRAF5* deficiency also exacerbates diet-induced obesity and metabolic disorders by promoting adipocyte inflammation in mice [47]. Nagashima et al. found that *TRAF5* restricts pro-inflammatory CD4 + T cell development by negatively regulating the IL-6 receptor signaling pathway [48].

To further explore the relationship between *TRAF5*, necroptosis, and smooth muscle cells in AS, we first validated *TRAF5* expression at the cellular and tissue level. We found increased *TRAF5* expression both after ox-LDL stimulation and in ASO tissue. To confirm this result, we performed *TRAF5* expression validation using multiple datasets containing carotid plaques, which were significantly lower. Previous studies have found that femoral plaques have higher plaque stability relative to carotid plaques due to their anatomical location and high fibrinogen content [49, 50], suggesting that higher *TRAF5* expression may be correlated to plaque stability. In vitro, *TRAF5* knockdown was followed by an increase in ox-LDL-induced necroptosis in smooth muscle cells, implying that low levels of *TRAF5* may lead to increased necroptosis within the plaque microenvironment, leading to plaque destabilization.

In advanced atherosclerotic lesions, reduced VSMC proliferation is detrimental to plaque stability [5], and human VSMCs from older vessels and advanced atherosclerotic plaques demonstrate reduced proliferation and prolonged population doubling time [51, 52]. Consistently, we found reduced proliferation in VSMCs after ox-LDL stimulation. Thus, in our current study, *TRAF5* not only serves as a prognostic marker for AS but also an indicator of plaque stability. Nevertheless, our study had certain limitations. First, bioinformatics mining requires a large number of clinical samples for further corroboration, and gene expression does not necessarily equate with protein expression. Second, the study of *TRAF5* requires further validation using animal models.

## Conclusions

In conclusion, our study identified *TRAF5* as a diagnostic marker of necroptosis-related atherosclerosis that can assess plaque stability. This finding may be helpful for the diagnosis of AS and the assessment of its stability.

## Supplementary Information

**Additional file 1: Supplementary Table 1.** Oligonucleotides used in this study. **Supplementary Table 2.** Primer sequence. **Supplementary Table 3.** GSEA data. **Supplementary Table  4.** GO&KEGG data.

**Additional file 2.**

## Data Availability

The original contributions presented in the study are included in the article/Supplementary Material; further inquiries can be directed to the corresponding authors.

## Abbreviations

TRAF5: TNF receptor associated factor 5
AS: Atherosclerosis
ASO: Arteriosclerosis obliterans
DEGs: Differentially expressed genes
NRDEGs: Necroptosis-related differentially expressed genes
LASSO: Least absolute shrinkage selection operator
RF: Random forest
ROC: Receiver operating characteristic
VSMCs: Vascular smooth muscle cells
ox-LDL: Oxidized low-density lipoprotein
RT-qPCR: Reverse transcription-quantitative polymerase chain reaction
GEO: Gene expression omnibus
KEGG: Kyoto encyclopedia of genes and genomes
GO: Gene ontology
NRGs: Necroptosis-related genes
GSEA: Gene set enrichment analysis
AUC: Area under the ROC curve
PPI: Protein–protein interaction
ASO: Arteriosclerosis obliterans
si-RNA: Small interfering RNA
TBST: Tris-buffered saline and tween-20
CCK8: Cell counting kit-8
