# Supplementary material for: Identification of necroptosis-related gene TRAF5 as potential target of diagnosing atherosclerosis and assessing its stability
Source: BMC Med Genomics. 2023 Jun 17;16:139. doi: 10.1186/s12920-023-01573-0 (PMC10276484; doi:10.1186/s12920-023-01573-0)
Supplement: Supplementary file 1 — Additional file 1: Supplementary Table 1. Oligonucleotides used in this study. Supplementary Table 2. Primer sequence. Supplementary Table 3. GSEA data. Supplementary Table 4. GO&KEGG data. [file 12920_2023_1573_MOESM1_ESM.docx]

[Supplementary Materials](javascript:;)

**Supplementary Table 1** Oligonucleotides used in this study

| Gene Name |  |
| --- | --- |
| si-*TRAF5*_1  si-*TRAF5*_2 | 5′-GAGAAGUCCUCAACUUAUA-3′ (Sense)  5′-UAUAAGUUGAGGACUUCUC-3′ (Antisense)  5′-CAACCUGUGCAGUGUUCUA-3′ (Sense)  5′-UAGAACACUGCACAGGUUG-3′ (Antisense) |

**Supplementary Table2** Primer sequence

| Gene Name |  |
| --- | --- |
| *TRAF5* | 5′-CAG TTG TTT GGC AAA AAT GGA-3′ (Sense)  5′-CAA TGT GAC TGG CAA AAA CC-3′ (Antisense) |

**Supplementary Table 3** GSEA data

| ID | Name | NES | p-value | FDR | q-value |
| --- | --- | --- | --- | --- | --- |
| hsa04620 | Toll-like receptor signaling pathway | 0.581979814 | 1.974224991 | 0.000625294 | 0.018272481 |
| hsa04621 | NOD-like receptor signaling pathway | 0.541297117 | 2.051858516 | 4.82E-05 | 0.003441431 |
| hsa05417 | Lipid and atherosclerosis | 0.501697662 | 1.96036874 | 0.000119009 | 0.003441431 |
| hsa04062 | Chemokine signaling pathway | 0.471452834 | 1.759494131 | 0.002613574 | 0.003441431 |

**Supplementary Table 4** GO&KEGG data

| Ontology | ID | Description | GeneRatio | BgRatio | pvalue | FDR | qvalue |
| --- | --- | --- | --- | --- | --- | --- | --- |
| BP | GO:0070266 | necroptotic process | 12/46 | 44/18670 | 2.08e-22 | 4.38e-19 | 2.34e-19 |
| BP | GO:0097300 | programmed necrotic cell death | 12/46 | 49/18670 | 9.04e-22 | 9.50e-19 | 5.08e-19 |
| CC | GO:0005741 | mitochondrial outer membrane | 7/46 | 178/19717 | 1.73e-07 | 1.37e-05 | 1.10e-05 |
| CC | GO:0046930 | pore complex | 4/46 | 23/19717 | 2.22e-07 | 1.37e-05 | 1.10e-05 |
| MF | GO:0005126 | cytokine receptor binding | 14/46 | 286/17697 | 9.11e-15 | 1.55e-12 | 1.09e-12 |
| MF | GO:0032813 | tumor necrosis factor receptor superfamily binding | 7/46 | 46/17697 | 2.47e-11 | 2.10e-09 | 1.48e-09 |
| KEGG | hsa04217 | Necroptosis | 46/46 | 159/8076 | 2.79e-82 | 4.38e-80 | 2.29e-80 |
| KEGG | hsa05164 | Influenza A | 23/46 | 171/8076 | 3.73e-27 | 2.93e-25 | 1.53e-25 |
