# Supplementary figures and images for: Identification of necroptosis-related gene TRAF5 as potential target of diagnosing atherosclerosis and assessing its stability
Source: BMC Med Genomics. 2023 Jun 17;16:139. doi: 10.1186/s12920-023-01573-0 (PMC10276484; doi:10.1186/s12920-023-01573-0)

The original uncropped images are below

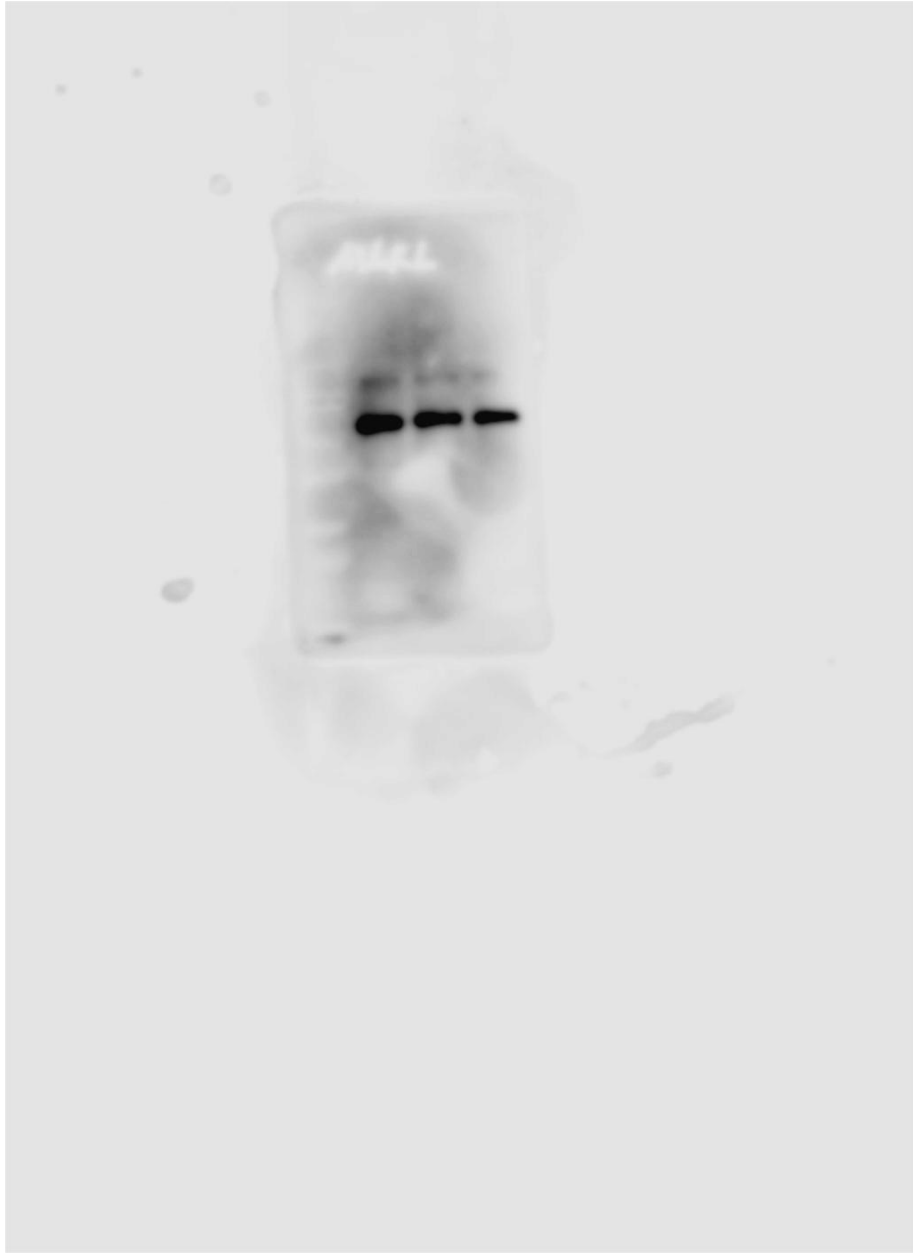

MLKL

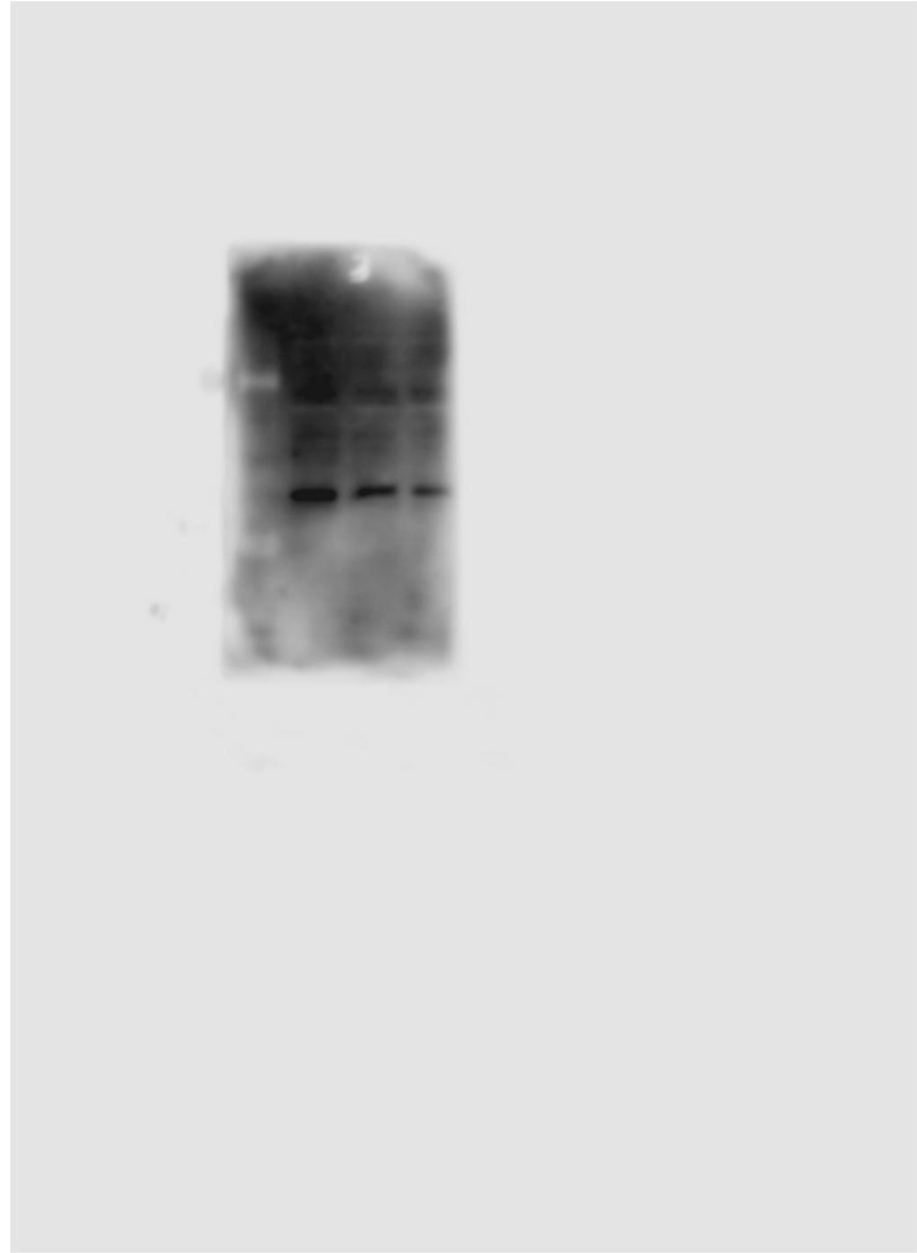

RIPK3

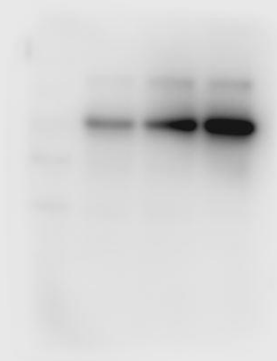

p-MLKL

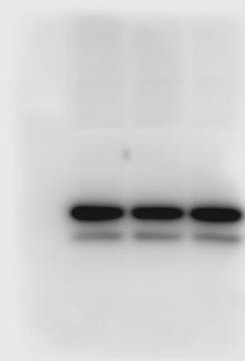

GAPDH

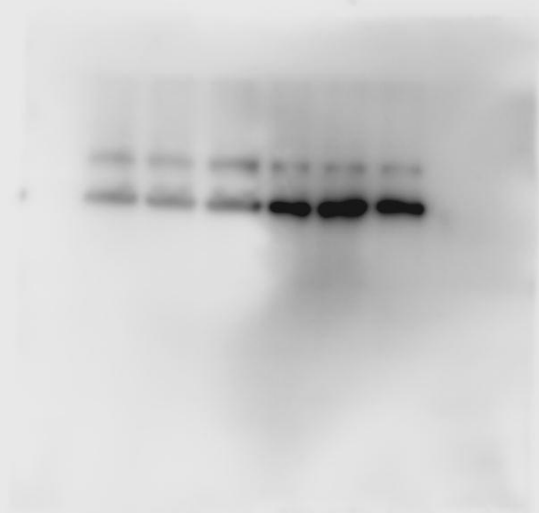

TRAF5

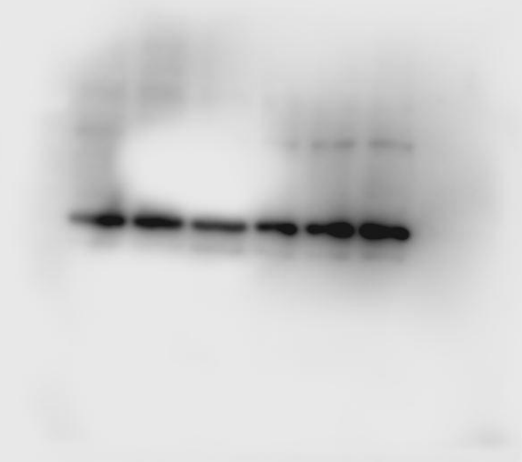

GAPDH

Supplement: Supplementary file 2 — Additional file 2. [file 12920_2023_1573_MOESM2_ESM.pdf]
